# A novel variant of the *SOX10* gene associated with Waardenburg syndrome type IV

**DOI:** 10.1186/s12920-023-01572-1

**Published:** 2023-06-26

**Authors:** Yanan Wang, Yuqiong Chai, Pai Zhang, Weiwei Zang

**Affiliations:** Luoyang Maternal and Child Health Hospital, Luoyang, 471000 China

**Keywords:** *SOX10* gene, Waardenburg syndrome type IV, Heterochromia iridis, Whole exome sequencing

## Abstract

**Background:**

Waardenburg syndrome (WS) is a rare genetic disorder characterized by varying degrees of sensorineural hearing loss and accumulated pigmentation in the skin, hair and iris. The syndrome is classified into four types (WS1, WS2, WS3, and WS4), each with different clinical phenotypes and underlying genetic causes. The aim of this study was to identify the pathogenic variant in a Chinese family with Waardenburg syndrome type IV.

**Methods:**

The patient and his parents underwent a thorough medical examination. We applied whole exome sequencing to identify the causal variant on the patient and other family members.

**Results:**

The patient presented with iris pigmentary abnormality, congenital megacolon and sensorineural hearing loss. The clinical diagnosis of the patient was WS4. The whole exome sequencing (WES) revealed a novel variant (c.452_456dup) in the *SOX10* gene, which could be responsible for the observed pathogenic of WS4 in this patient. Our analysis suggests that this variant produces a truncated protein that contributes to the development of the disease. The genetic test confirmed the diagnosis of WS4 in the patient from the studied pedigree.

**Conclusions:**

This present study demonstrated that genetic test based on WES, an effective alternative to regular clinical examinations, helps diagnose WS4. The newly identified *SOX10* gene variant can expand the understanding of WS4.

## Background

Waardenburg syndrome (WS) is a congenital disorder characterized by heterochromia iridis and congenital deafness [1]. It occurs with a frequency of 1 in 42,000 and contributing to 2–5% of congenital deafness cases. The syndrome follows an incomplete dominant pattern of inheritance [2]. The main clinical features in patients with Waardenburg syndrome are congenital sensorineural hearing loss, dystopia canthorum, and pigment disturbances of hair, skin and iris [3].

The known genes associated with Waardenburg syndrome are mainly localized on chromosomes 2, 3, 8, 12, 13, 20 and 22; their associated genes are *PAX3*, *MITF*, *SNAI2*, *KITLG*, *EDNRB*, *EDN3*, and *SOX10*. WS1 and WS3 are predominantly caused by *PAX3* variants, typical clinical features are ectopic endophthalmos, asymmetric hearing loss, skin depigmentation and gray hair on the forehead [4]. Sensorineural deafness is present in 47–53% of WS1 patients, iris heterochromia in 15–30% of cases, nasal root widening in 52–100% of cases and connected eyebrows in 63–73% of cases [5]. WS3 is similar to WS1 with presence of upper limb abnormalities in addition, such as joint contractures, skeletal hypoplasia of the upper extremities, short fingers, and finger contractures. The causative genes of WS2 are complex, and the causative genes are still not identified in more than 50% of patients. Based on the different causative genes, they are further classified into five types: 2 A, 2B, 2 C, 2D and 2E. In WS2, congenital inner ear malformations are mostly seen in patients with *SOX10* variants, while facial freckles are the main feature of patients with *MITF* variants [4, 6]. Patients with WS2 exhibit sensorineural deafness in 87% of cases [7]. WS4 is further classified into 4 A, 4B, and 4 C types depending on the causative gene, with the *EDNRB* variant predominant in type 4 A, the *EDN3* variant in type 4B, and the *SOX10* variant in type 4 C. The *SOX10* variant are the most common cause. WS4 also called Shah-Waardenburg syndrome or Waardenburg-Hirschsprung disease, is present with Hirschsprung disease which manifests in severe congenital constipation or lifethreating intestinal obstruction resulting from aganglionosis of a part or all of the colon [8–10]. However, the clinical manifestations of Waardenburg syndrome do not always correspond precisely to the causative gene, and the clinical phenotype may differ between individuals in the same family due to differences in penetrance or other factors such as age and environment. Additionally, not all individuals with the syndrome will display all of its manifestations.

The *SOX10* gene (SRY- (sex-determining region Y-) box10) is located on chromosome 22q13.1 and belongs to the *SOX* (SRY-related HMG-box) family of transcription factors. It contains five exons and encodes a protein which consists of 466 amino acids. *SOX10* is involved in many cellular developmental processes,it is first expressed in the dorsal neural tube at the early stage of neural crest cells (NCC) migration. With differentiation of the NCC, *SOX10* begins to be widely expressed throughout the adult body, such as in the hair follicles, inner ear, iris, and gastrointestinal tract [11]. *SOX10* is a transcription factor for the *MITF* gene, and MITF plays a crucial role in the development of melanocytes. The etiology of WS is currently believed to be caused by the abnormal growth of NCC [12, 13]. In addition, the hearing loss in WS may be associated with abnormal proliferation, survival, differentiation, or migration of NCC-derived melanocytes [11]. Genetic variants that affect melanocyte differentiation and migration may also affect the intracochlear potential, leading to sensorineural deafness [14]. Due to the significant genetic heterogeneity of WS, it is essential to further understand its molecular mechanisms and gather more data on the gene variants that cause it. In this report, a novel variant (c.452_456dup) of the *SOX10* gene was detected in a Chinese family with WS4. The discovery of this study may provide valuable information for genetic counseling of WS4 families.

## Methods

### Family description

The patient, an 8-year-old boy presented with congenital deafness, hirschsprung, and bilateral pale blue irises, the clinical diagnosis of the patient was Waardenburg syndrome type IV. Neither of the boy’s parents exhibited similar symptoms. The patient underwent a complete physical examination, clinical audiological evaluation (including pure tone audiometry, acoustic conductance, auditory brainstem response, steady-state auditory evoked potentials, and aberrant products of otoacoustic emissions), laboratory tests, medical history and family history inquiries. Additionally, 5 mL of peripheral venous blood was collected from the patient and his parents for genomic DNA preparation and candidate variant screening. The study was approved by the the Ethics Committee of Medical Genetics and Prenatal Diagnosis of Luoyang Maternal and Child Health Hospital, and the parents of the patient signed an informed consent form.

### Genomic DNA extraction and whole exome sequencing

The peripheral venous blood genomic DNA was extracted by TIANamp genomic DNA extraction kit (Tiangen Biotech, China) in strict accordance with the manufacturer’s protocol. The genomic DNA concentration and purity were detected by dsDNA BR Assay Kit (Thermo Fisher Scientifific, USA). All peripheral venous blood genomic DNA samples were diluted to 10 ng/µl, stored at − 20 ℃.

WES was performed by Annaroad Gene Technology (Beijing) Co. using SureSelect (V5 + UTR; Agilent) for target capture (100 bp Pair End mode and 100x coverage). The quality of the data was assessed by FastQC and the data was processed following Broad Institute’s best practice guidelines for GATK v3.4 (https://www.broadinstitute.org/).

We use databases such as “Varcard, PolyPhen2, Mutation Taster, SIFT (Sorting intolerant from tolerant)” and other similar tools to screen for pathogenic genes. The predicted pathogenic loci are considered disease related candidate loci, and the “Exomiser” software is used to screen for pathogenic loci in combination with clinical phenotypes.

### Sanger sequencing validation

We designed and synthesized PCR primers for sequencing based on the variant sites obtained from the whole exome assay, amplified the DNA, purified the products, and performed Sanger sequencing using ABI sequencer. The SOX10 forward primer is CCTAGAGTCCAGGGTCTCATTGC and the reverse primer is CCAGGGCCTCACATCTTCCAAG. The amplification consisted of an initial denaturation stage at 94 °C for 3 min, followed by 35 cycles consisting of denaturation at 94 °C for 30s, annealing for 30s at 60 °C, and extension at 72 °C for 50 s, with an extension step performed at 72 °C for 3 min. The “Mutation Surveyor” analysis software compares the sequencing results with the reference sequences.

### Prediction of the secondary structure of SOX10 (Mut: p. F153fs *135)

The PSIPRED Workbench is a web server offering a range of predictive methods to the bioscience community. We extract the homologous sequence of the human SOX10 protein from the NCBI-Homologene Database and input the variant sequence on the online website PredictProtein (http://www.predactprotein) [15] to predict the secondary structure of SOX10 (Mut: p. F153fs *135).

## Results

### Clinical symptoms

**Fig. 1:**
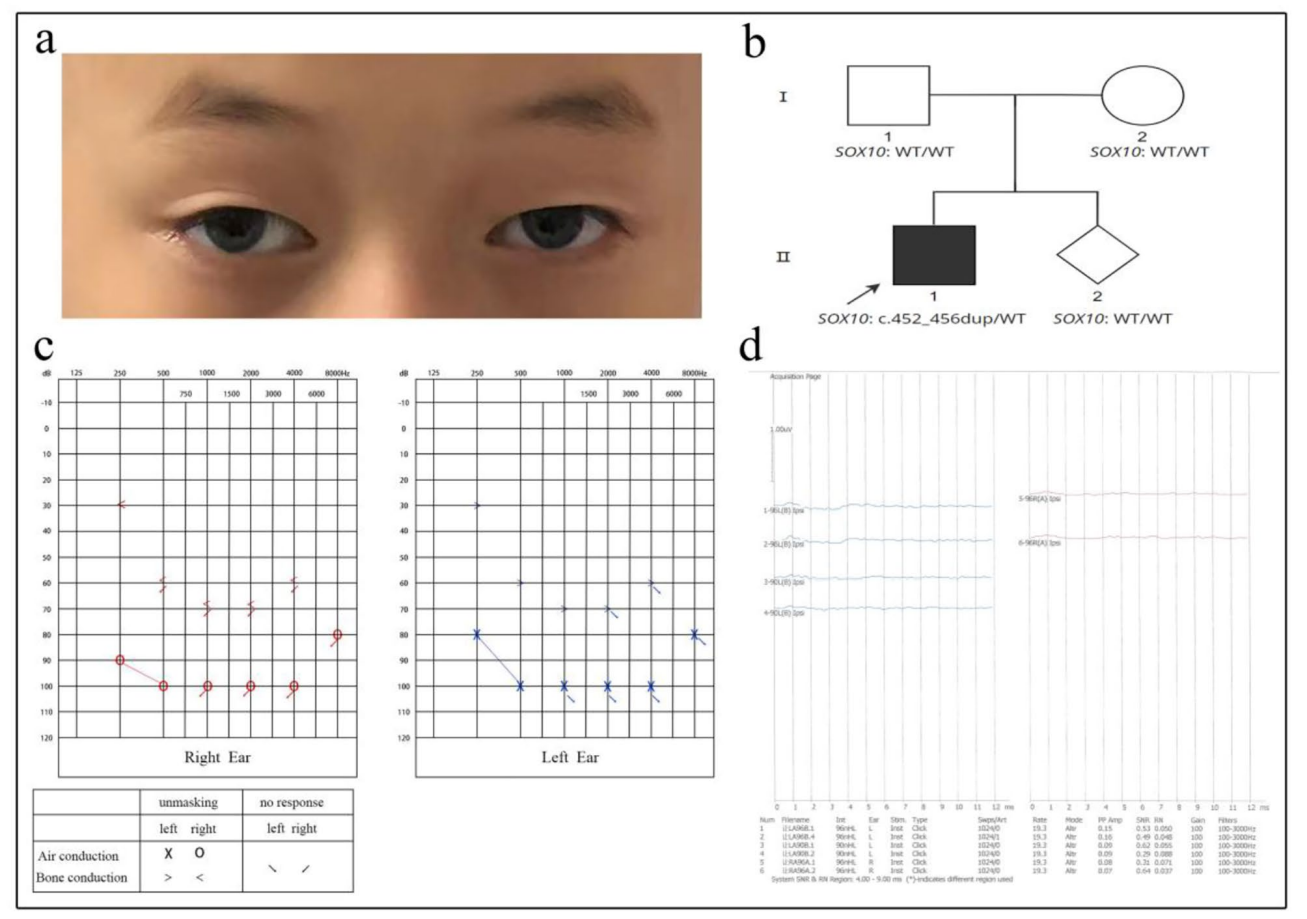
Clinical features and Family pedigree of the patient. (a) The iris heterochromia in both eyes of the patient, which are blue. (b) The pedigree indicates that Family II-1 had a spontaneous heterozygous variant (*SOX10*:c.452_456dup), which is marked black. No variants were found in any other members of the family. (c) and (d) is the clinical audiology examination of the patient. (c) ASSR (auditory steady-state responses) of the right ear: 90 dB and 100 dB at 250 and 500 Hz; ASSR of the left ear: 80 dB and 100 dB at 250 and 500 Hz; the remaining frequencies showed no response. (d) present brainstem auditory evoked potential. The patient exhibit severe hearing impairment (no identifiable wave at 96 dB nHL and 90 dB nHL in pure-tone audiogram; no detection of binaural sound emission in brainstem auditory evoked potential graph)

This patient was an 8-year-old boy, he was the first child of a non-consanguineous couple, he had congenital deafness, bilateral pale blue iris (Fig. 1a), sensitivity to bright light, mild facial hyperpigmentation, normal vision, and average intelligence. A congenital megacolon was found after birth and was surgically removed. There was no history of asphyxia, hypoxia or neonatal jaundice at birth; no history of maternal medication or radiation exposure during pregnancy; no history of ototoxic medication; no oral abnormalities; no abnormalities in the skeletal joints of the extremities, chest and reproductive organs. The family pedigree of the patient is shown in Fig. 1b. The audiology examination of the patient showed failed bilateral otoacoustic emissions; ASSR (auditory steady-state responses) showed the thresholds of the right ear was 90 dB nHL at 250 Hz, 100 dB nHL at 500 Hz, while the thresholds of the left ear was 80 dB nHL at 250 Hz, 100 dB nHL at 500 Hz, the remaining frequencies showed no response. There also no identifiable wave at 96 dB nHL and 90 dB nHL in pure-tone audiogram; no detection of binaural sound emission in brainstem auditory evoked potential graph (Fig. 1c and d). According to a previous report, in the appropriate clinical context, bilateral agenesis or hypoplasia of the semicircular canals or both, associated with an enlarged vestibule and cochlear deformity, strongly suggests a diagnosis of WS linked to a SOX10 variant. Unfortunately, the patient’s parents refused this examination. Based on the hearing examination, the patient was diagnosed with sensorineural deafness in both ears. The patient’s communication in daily life was essentially normal. The patient’s parents were clinically phenotypically normal, with no hearing impairment or iris heterochromia. There were two pregnancies and one full-term birth for the mother. At this point in her pregnancy, at 23 weeks, a prenatal diagnostic fetal examination has shown no abnormalities.

### SOX10 variant analysis

Both the patient and his parents underwent whole exome sequencing tests. The WES results were compared with the human reference genome (GRCh37/hg19). The patient carried a heterozygous variant: the SOX10 c.452_456dup (NM_006941); the c.452_456dup variant located at exon 3 of *SOX10*. This variant involves a 5-base repeat, leading to a shift in the amino acid code and resulting in an early stop codon at position 152 followed by 135aa. As a result, truncated proteins that can only encode 286 amino acids were produced, leading to a loss of normal function of the *SOX10* protein. No variants were detected in other genes, and no genetic variants were identified in the patient’s parents.

**Fig. 2:**
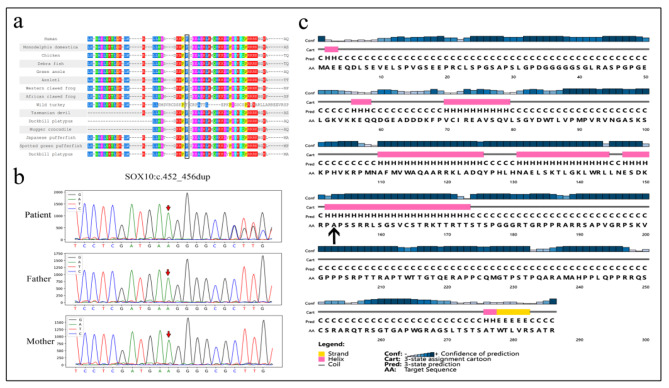
(a) Amino acid conservativeness analysis. Shown are 75 amino acids surrounding the variant position (marked with a black box) The sequence is highly conserved in several species. The amino acid where the variant c.452 456dup p.F153fs*135 is found is highly conserved and is phenylalanine in all 14 species, according to PolyPhen-2 analysis. (b) Sanger sequencing map of the patient and his father and mother. The patient has the *SOX10*: c.452_456dup heterozygous variant. Both the patient’s father and mother have no variant at this location. (c) Secondary structure of sox10 (p.F153fs*135), and arrow indicated variant position

We also searched the relevant databases, and no report related to the c.452_456dup variant in *SOX10* was found in the Human Gene Mutation Database (HGMD), Human Exome Database (ExAC), ClinVar database, or the Genome Aggregation Database (gnomAD), and it was considered to be a de novo variant. Then,we did a functional analysis of the variant protein. Using the PolyPhen-2 (Polymorphism Phenotyping v2) to estimate the sequence’s conservatism, the sequence was compared to those of several different species, showing a high level of conservatism (Fig. 2a). It indicates that this locus has a potential functional impact. Referring to the American College of Medical Genetics and Genomics (ACMG) gene mutation interpretation guidelines, this locus is consistent with four lines of evidence (PVS1_Strong, PS2, PM1, PM2_Supporting) and is graded as Pathogenic.

The variant of the *SOX10* gene were then confirmed by sanger sequencing. From the sequencing results, we could see that the variant c.452_456dup of the patient was de novo (Fig. 2b), consistent with the whole exome sequencing results. It was discovered that neither the mother nor the father had the variant at the same locus, and the test result was wild-type, patient with heterozygous variant consistent with WS4 dominant pattern of inheritance.

The secondary structure of *SOX10* (p.F153fs*135) is shown in Fig. 2c. Because of the *SOX10*:c.452_456dup variant, the amino acid at position 153 changes from phenylalanine to alanine. The secondary structure of proteins serves as the foundation for proteins to create tertiary structures and carry out regular functions. We could see that the Pro153 residue was involved in the coil of the secondary structure and the prediction reliability was high. It was a highly conserved residue located on a conserved secondary fragment. The human SOX10 protein’s ability to perform its function may be impacted by variant in this residue, which could ultimately result in illness.

## Discussion

Waardenburg syndrome is a complex syndrome with different clinical phenotypes in different patients. It is characterized by clinical manifestations of oculocutaneous anomalous pigmentation, deafness of varying degree, dystopia canthorum and broad nasal root.

In this study, we report a case diagnosed with WS4 that presented with abnormal iris pigmentation, congenital megacolon and sensorineural deafness. We found the heterozygous variant c.452_456dup (p.F153fs) in exon 3 of the *SOX10* gene in the patient, which was not reported by the HGMD and was considered a de novo variant. Still, the possibility of germline chimerism could not be excluded entirely. It can guide us in subsequent pregnancies of the couple, where targeted genetic testing can be offered to diagnose potentially affected fetuses.The variant is located in exon 3 and can cause protein p.F153fs, leading to premature termination of translation and causing a change in protein conformation.

According to the current research on the *SOX10* variant causing WS, the study of its protein molecular signaling mechanism is slow due to the lack of adequate animal models. However, researchers have still predicted its pathogenesis, mainly including the following three: (1) haploinsufficiency effect: This mechanism suggests that the wild, unmutated protein may fill the impaired protein function produced by the mutated gene, but the difference in the amount of normal protein that can remain in different individuals and the amount of protein required by individuals may result in different severity of clinical symptoms in different individuals [16–18]. (2) Dominant negative effect: This mechanism predicts that if one of the two alleles is mutated, even if the mutated gene loses all function, the other wild-type gene should still retain 50% of its function. However, in some physiological conditions, the mutated protein or gene not only loses its normal function but also inhibits the activity of the wild-type protein. This phenomenon of proteins interfering with each other is called the dominant negative effect. It is commonly believed that WS4 may have a series of severe peripheral nerve or digestive tract dysfunctions due to this effect [19–21]. (3) Functional redundancy: This mechanism suggests that *SOX10* is derived from the SOX-E group of the SOX superfamily, including *SOX8*, *SOX9*, and *SOX10*, which are functionally complementary and very similar, so that when a variant in *SOX10* results in the lack of function or complete absence of its encoded protein, *SOX8* or other members of the SOX family could potentially fill or replace the former function and thus exhibit less severe clinical symptoms [22, 23]. In this study, we detected a de novo variant in the SOX10 gene, and we hypothesize that this variant caused a dominant-negative effect, which in turn led to the severe phenotype of WS4.

However, due to the clinical diversity and genetic heterogeneity of WS, there is no fixed relationship between clinical phenotype and genotype, and it is difficult to explain the clinical phenotype by one mechanism. The current research on the pathogenesis of WS is still based on in vitro molecular experiments, which cannot fully simulate and reflect the pathogenesis of the organism. For example, regarding the role of transcriptional regulation followed by translational modifications in the regulatory mechanisms of neuronal cell growth, we do not know what role *SOX10* or other WS-related genes have in these processes. Therefore, these are intriguing inquiries that might offer concepts and guidelines for future research on the pathogenesis of WS.

## Conclusions

We identified a novel variant site in the SOX10 gene in WS4 for the first time in a Chinese family, which may constitute a candidate pathogenic variant associated with WS4. At the same time, our new finding expanded the database of WS pathogenic gene variants, facilitating accurate diagnosis and genetic counseling for heterogeneous WS4.

## Data Availability

The datasets generated and analysed during the current study are available in the Genome Sequence Archive for Human repository, and accession number to datasets is HRA004237. It can be accessed from the following link:https://bigd.big.ac.cn/gsa-human/browse/HRA004237.

## Abbreviations

WS: Waardenburg syndrome
*SOX10*: SRY-box transcription factor 10
WES: Whole exome sequencing
NCC: Neural crest cells
PCR: Polymerase chain reaction
